# The nature of rehabilitation services provided to children with cerebral palsy: a population-based nationwide study

**DOI:** 10.1186/s12913-019-4111-4

**Published:** 2019-05-02

**Authors:** Seong Woo Kim, Ha Ra Jeon, Taemi Youk, Jiyong Kim

**Affiliations:** 1 Department of Physical Medicine and Rehabilitation, National Health Insurance Service Ilsan Hospital, 100 Ilsan-ro Ilsan-donggu, Goyang-si, Gyeonggi-do 10444 South Korea; 2 Department of Statistics, Korea University, 145 Anam-ro, Seongbuk-gu, Seoul, 02841 South Korea; 3 Department of Physical Medicine and Rehabilitation, Inje University Ilsanpaik Hospital, Goyang, South Korea; 4 Yonsei University College of Medicine, 50-1 Yonsei-ro, Seoul, Seodaemun-gu 03722 South Korea

**Keywords:** Cerebral palsy, Rehabilitation, Botulinum toxins, national health programs

## Abstract

**Background:**

Cerebral palsy (CP) is a serious neurodevelopmental disorder that occurs in childhood and requires a range of treatments over a person’s lifetime. The aims of this study were to investigate the nature of the rehabilitation treatments provided to children with CP and to determine if there were any changes in patterns over time.

**Methods:**

From 2003 to 2013, the nature of rehabilitation treatment was analyzed for children diagnosed with CP. In addition, the medical data of rehabilitation treatments over a 10-year period (from birth to nine years of age) were analyzed for children born in 2004 diagnosed with CP. Furthermore, we analyzed whether there was a difference in the costs of medical expenditures according to family income. All studies were based on data from the Korean National Health Information Database.

**Results:**

It was found that, in recent years, rehabilitation therapy and spasticity treatment of children with CP have started being performed at a younger age than in the past. Among the children with CP born in 2004, 28.6% had physical therapy and 25.4% had occupational therapy on an inpatient basis; 81.3% had physical therapy and 62.2% had occupational therapy on an outpatient basis. Additionally, 22.2% of children received botulinum toxin injection therapy at least once. The numbers of children receiving rehabilitation therapy and botulinum toxin injection were highest at 1–5 years of age and 6–7 years of age, respectively. The expenditure on rehabilitation therapy was not affected by the economic level of the family.

**Conclusion:**

This study investigated the nature of rehabilitation services provided to children with CP. More recently, the treatment of children with CP has started to be performed earlier than in the past. In addition, it was confirmed that the nature of rehabilitation treatment for children with CP changed according to age. Based on these results, services and health policies may need to be better organized to enhance the benefits to children with CP.

## Background

Cerebral palsy (CP) is a neurodevelopmental disorder that affects movement and posture due to nonprogressive damage to the immature brain, ultimately affecting the child’s lifespan [1]. CP changes in severity and functional level as the child grows and develops into an adult. The mobility performance that children can eventually reach is dependent on their functional level when they are young, so optimized therapies must be continuously provided to develop functional levels to their maximum potential and prevent functional deterioration from occurring [2]. Various therapies have been revealed to be effective for children with CP [3]. Examples of the therapies that are used in Korea include physical therapy, occupational therapy, hydrotherapy, and botulinum toxin injections (BTx.). According to unpublished results of work undertaken by the authors regarding rehabilitation treatment for children with CP in Korea over the past decade, the proportion of children receiving outpatient treatment was higher than inpatient treatment. Further, orthopedic surgery for optimization of musculoskeletal function was most commonly undertaken in school-aged children. Outpatient rehabilitation treatment typically would be continued following hospitalization for intensive therapy after orthopedic surgery. There are significant medical and social expenses associated with the various therapies and specialized care for children with CP. In the United States, the lifetime cost for a child with CP is reported to be 11.5 billion dollars [4]. In Denmark, 40,265 euros per year is spent per a child with severe CP [5]. In a few studies published in Korea regarding the cost of treatment for children with CP, Park et al. [6] found that one person with CP spends 1.8 times more on lifetime medical expenses than an equivalent person without a developmental disability. A higher occurrence of unmet health care needs among children with special health care needs has been identified in various studies. Using the 2007 National Survey of Children’s Health, the authors found that about 4% of US children have unmet medical care needs [7, 8]. McManus et al. reported that children who need special health care needs have an unmet rehabilitation need and there was a difference in the therapeutic use according to severity of developmental condition [9, 10]. Jackson and colleagues reported that children with CP have more unmet needs within the children with special health care needs due to the complexity and potential severity of the disability [11].

Korea operates the National Health Insurance system, which is compulsory and required by Korean law for all citizens to enroll in. The National Health Insurance Service, NHIS, is the only public medical insurance institution operated by the Ministry of Health and Welfare in Korea. The NHIS provides universal coverage to all Korean nationals with the exception of the lowest-income group, which is supported by the Medical Aid program. Benefits are provided for all aspects of healthcare including rehabilitation treatment, with diagnosis, drugs, therapies and surgery also being included. Rehabilitation therapy includes physical therapy, occupational therapy, and hydrotherapy; these therapies have been proven to be effective for children with CP and are covered under the healthcare benefits. Since 2005, the cost of BTx. is covered by insurance for children with CP if its use meets certain criteria. In Korea, rehabilitation treatment is administered on either an inpatient or outpatient basis, and a large part of treatment of children with CP is covered by the National Health Insurance. Therefore, it is possible to analyze the nature of rehabilitation services provided to children with CP based on the content of service requests to the NHIS. Financial revenue of the National Health Insurance of Korea consists of contributions of the insured and government subsidy. Under National Health Insurance, the contributions differ according to the family income level; the higher the income is, the greater the contribution must be. While the NHIS is publicly funded, additional expenditures are required of the user. A person who receives healthcare service must pay certain portions of the total healthcare expenses as ‘copayments’. The amount of the copayment differs according to the level of healthcare institutions and outpatient/inpatient services, and ranges from approximately 20–60%. The treatment cost described in this study is the sum of the NHIS burden and the copayment burden of the individual receiving the treatment.

The National Health Information Database, NHID, was formed by the NHIS and is a public database on health care utilization, health screening, sociodemographic variables and mortality for the whole population of South Korea [12]. We thought that identifying the factors that affect the use of rehabilitation treatment and the patterns of the use of rehabilitation in children with CP could be the basis for providing more appropriate treatment. The purpose of this study was to investigate the change in rehabilitation treatment over key age periods in children with CP and the difference in age-related rehabilitation treatment based on data held by the NHID. Additionally, this study investigated the difference in rehabilitation cost according to family economic status.

## Methods

### Inclusion criteria

**Fig. 1:**
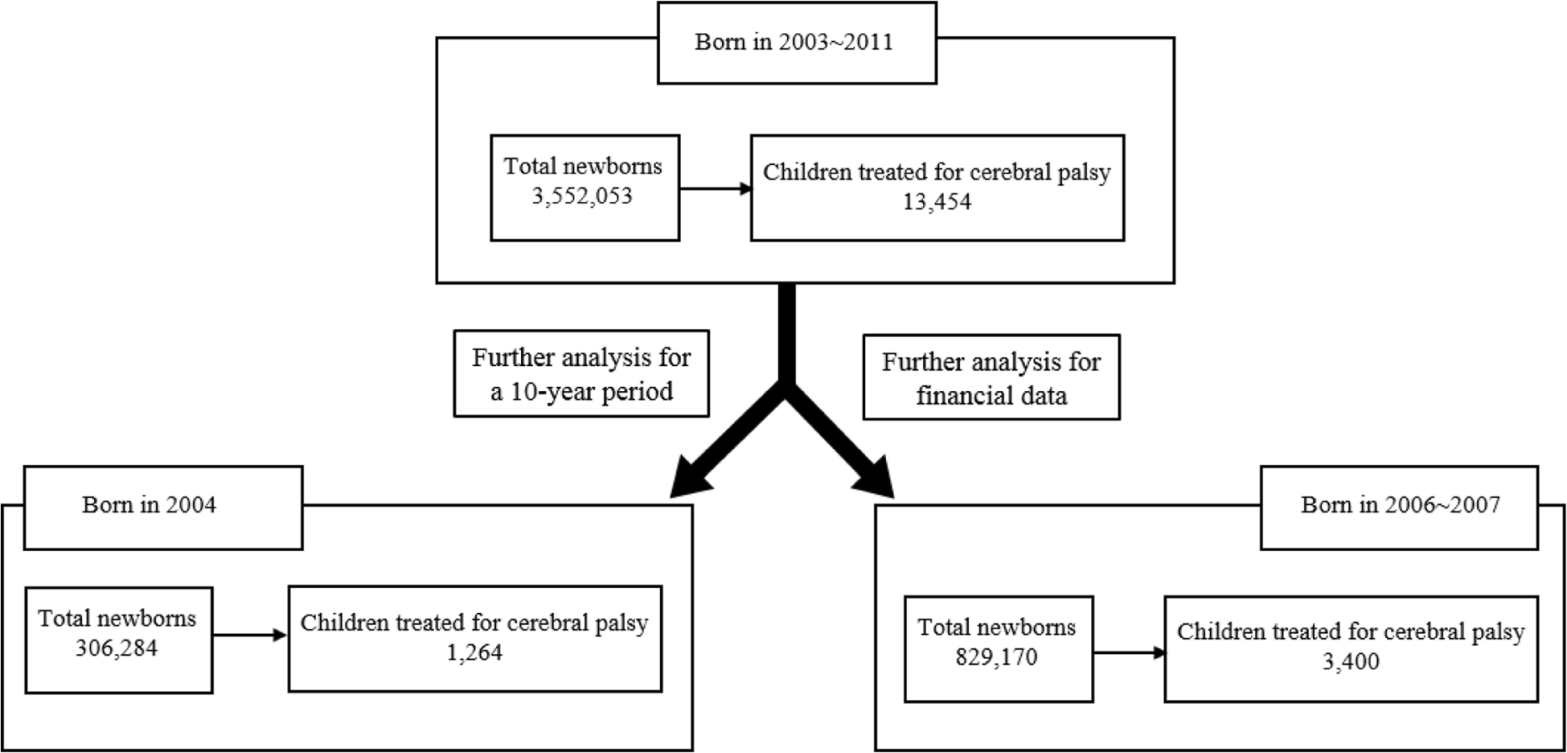
Flow sheet for Inclusion Samples (unit: n)

This study was based on 11 years of data from the NHID, from January 1, 2003 to December 31, 2013. Data on children who had more than two medical claim records related to a diagnosis of CP after 24 months of age within this period were analyzed in the study. According to the ICD-10 (International Classification of Disease, 10th edition), CP-related codes are spastic CP (G800–802, G810, G811, G819, G830–833); dyskinetic CP (G803); ataxic CP (G804); and unspecified CP (G808). We analyzed data of treatment claims for newborns born between 2003~2011 and diagnosed with CP (Fig. 1).

### Further analysis of a 10-year period

We also analyzed the changes in the nature of rehabilitation services provided to children with CP born in 2004 and followed for a 10-year period (ages 0–9 years). Rehabilitation treatments including physical therapy, occupational therapy, hydrotherapy, and BTx. were analyzed for the corresponding procedure codes or corresponding drug code. Data on orthopedic surgery and neurosurgery for children with CP were also considered but were ultimately excluded from the analysis due to the small number of cases.

### Further analysis for financial data

We collected information about the amount of financial contribution to see if there were any differences in treatment cost based on family economic status. For the statistical analysis, the amount of contribution was divided into five groups; the first group included the Medical Aid and the lowest-income group and then progressively higher income groups were included up to the fifth group. Contribution information related to children born in 2004 was often missing; thus, the difference of treatment cost according to the economic status was analyzed based on children born in 2006–2007 to ensure the accuracy of the information.

### Ethics approval

Ethics approval was obtained following project approval by the National Health Insurance Service Ilsan Hospital (NHIMC 2015–02-015). The need for written consent was formally waived by the ethics committee.

### Statistical analysis

SAS software (ver. 9.4; SAS Institute Inc., Cary, NC) was used for all statistical analyses. The Kruskal-Wallis statistic was used to examine the difference in treatment cost for income. The level of statistical significance was set at *p* < 0.05.

## Results

### Change in the age at which treatment began

Of the 3,552,053 newborns born between 2003 and 2011, a total of 13,454 children were treated for CP. Analysis of the starting age of the rehabilitation therapy such as physical therapy or occupational therapy in the form of outpatient or inpatient treatment showed that recently, children started rehabilitation therapy earlier than before. In the case of children born in 2003, rehabilitation therapy started at an average age of 2.1 ± 2.0 years, whereas in the case of children born in 2011, it was found that the therapy started at an average age of 1.0 ± 0.7 years.

**Fig. 2:**
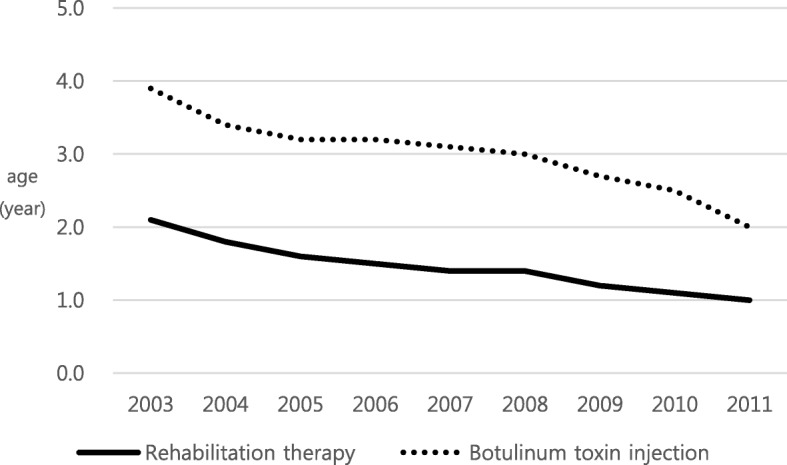
Change in the Age at which Treatment Began according to Birth Year (unit: year)

A similar trend was observed with BTx., i.e., children born in 2011 started BTx. at an average age of 2.0 ± 0.1 years, which was earlier than that for children born in 2003, who started at an average age of 4.0 ± 1.5 years (Fig. 2).

### Comparison of rehabilitation therapy according to age

**Fig. 3:**
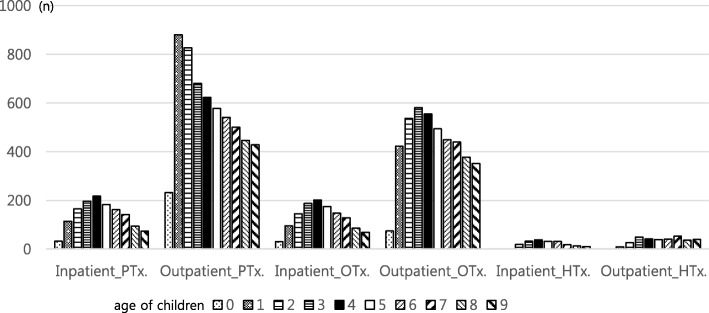
Number of Patients receiving Rehabilitation Therapy according to Age (unit: n)

Of the 306,284 newborns born in 2004, a total of 1264 children were treated for CP. We reviewed the rehabilitation therapy data of these children with CP up to 9 years of age. Among the children with CP, 28.6% had undergone physical therapy, 25.4% had occupational therapy, and 7.0% had undergone hydrotherapy on an inpatient basis. Overall, 81.3% of all children with CP had physical therapy, 62.2% had occupational therapy and 8.9% had hydrotherapy on an outpatient basis (Table 1). When the number of children treated by age was examined, the number of children receiving all rehabilitation therapies was highest in the 1- to 5-year age and subsequently decreased as age increased. In particular, the number of children receiving all rehabilitation therapies between 4 and 5 years old was the highest. In the case of outpatient physical therapy, the number of children receiving the therapy was highest at the age of 1 year. In the case of hydrotherapy, there was no consistent increase or decrease in the pattern according to age, but its use remained within a certain range after the child was 3 years old (Fig. 3).

**Table 1:** General Baseline Characteristics of the Children with Cerebral Palsy Born in 2004

Variables		Number of children (%)
Sex	Female/Male	533/731 (42.2/57.8)
Inpatient basis	Physical therapy	362 (28.6)
	Occupational therapy	321 (25.4)
	Hydrotherapy	88 (7.0)
Outpatient basis	Physical therapy	1028 (81.3)
	Occupational therapy	786 (62.2)
	Hydrotherapy	112 (8.9)
Botulinum toxin injection		281 (22.2)

**Fig. 4:**
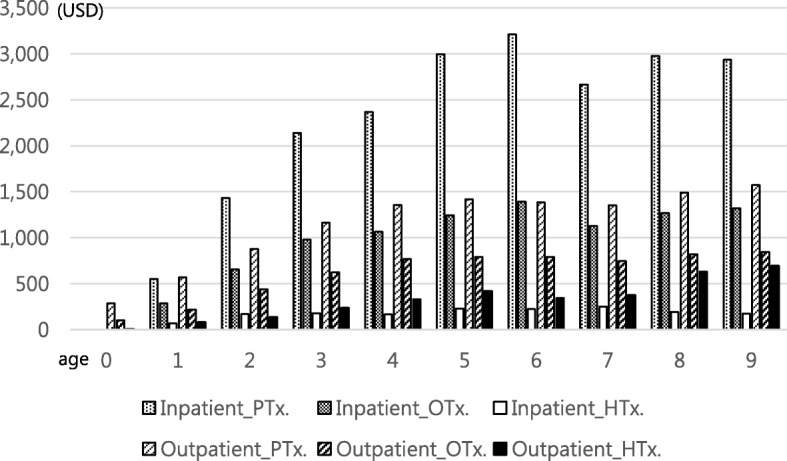
Cost of Rehabilitation Therapy per person according to Age (unit: USD)

**Fig. 5:**
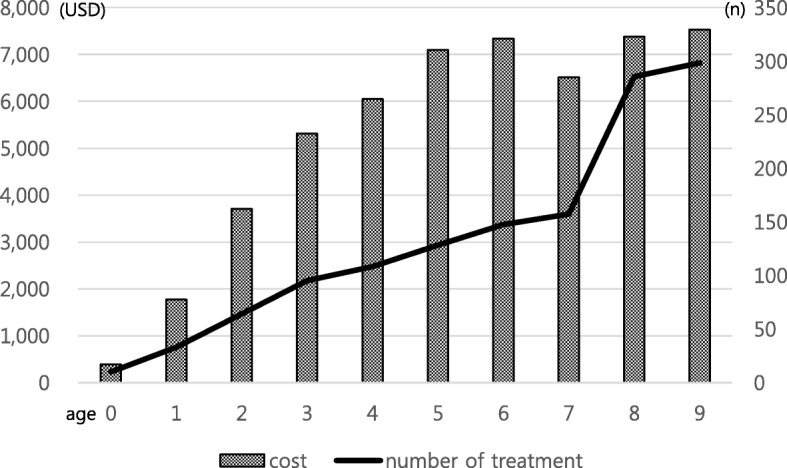
Total Cost and Number of Treatment per person according to Age (unit: USD, n)

Additionally, the overall cost of rehabilitation therapy per person increased with age (Fig. 4). These costs averaged USD 1949 for inpatient physical therapy and USD 856 for inpatient occupational therapy per year for the last 10 years. The average cost of outpatient physical therapy was USD 1051, and the average cost of outpatient occupational therapy was USD 562 per year. The average cost of inpatient hydrotherapy was USD 152, and the average cost of outpatient hydrotherapy was USD 299 per year. When the per capita therapy expenditure over 10 years was examined, the expenditure for therapy gradually increased until six years of age, after which a similar level was maintained. Furthermore, the number of therapies per person was observed to increase steadily with age (Fig. 5).

### Changes in botulinum toxin injection

Data of BTx. therapy in children born in 2004 with CP were analyzed up to the age of 9 years. Of the total 1264 children, 281 (22.2%) received BTx. therapy at least once (Table 1). The number of BTx. treatments per person was the highest between 6 and 7 years of age and decreased thereafter. When the figures for BTx. treatment for children with CP born in 2007 were compared, the frequency was 1.8 times higher than that of children born in 2004. The number of BTx. treatments per person for children born in 2004 increased sharply at 6 years of age, while that for children born in 2007 increased at the age of 3 years (Table 2).

**Table 2:** Comparison for Botulinum Toxin Injection Treatment

	Number of BTx. treatments per person	
Age	2004	2007
Birth year		
0 ≤ year< 1	0	0
1 ≤ year< 2	1.00	1.50
2 ≤ years< 3	1.30	1.27
3 ≤ years< 4	1.32	2.26
4 ≤ years< 5	1.37	2.43
5 ≤ years< 6	1.40	2.39
6 ≤ years< 7	2.36	2.19
7 ≤ years< 8	2.39	–
8 ≤ years< 9	1.97	–
9 ≤ years< 10	1.82	–

BTx., botulinum toxin injection

### Difference in rehabilitation therapy cost based on economic levels

We analyzed the total cost of rehabilitation therapy over 7 years for children diagnosed with CP born between 2006~2007 and tried to determine if there were any differences dependent on the economic status of the family. Total rehabilitation therapy cost was defined as the sum of physical therapy, occupational therapy and hydrotherapy on either an inpatient or outpatient basis. The 1st group spent USD 8274 for the total cost of rehabilitation therapy per person for 7 years while the 2nd, 3rd, 4th and 5th groups spent USD 7376, USD 7238, USD 7047 and USD 7123, respectively. The 1st group included the Medical Aid and the lowest income group and then progressively higher income groups were included up to the 5th group. The results showed no significant differences in rehabilitation expenditure between the different income levels (*p* = 0.993).

## Discussion

Cerebral palsy is characterized by movement and posture abnormalities accompanied by secondary complications such as joint contracture and postural deformity [13] and requires various treatments over an extended period of time. A higher occurrence of unmet health care needs among children with special health care needs including CP has been identified in various studies [7–11]. We thought that understanding the state of care provided to children with CP would be the basis for solving unmet health care needs. So we conducted a large-scale study based on the Korean national database of the NHID to investigate the nature of rehabilitation services provided to children with CP and see if there had been changes in patterns over the past 10 years. It was found that rehabilitation therapy and BTx. are being performed at a younger age in Korea compared with the past. From 0 to 9 years of age, the numbers of children receiving rehabilitation therapy and BTx. were highest at 1–5 years of age and 6–7 years of age, respectively. The expenditure on rehabilitation therapy was not affected by the economic level of the family.

The usefulness of early diagnosis and early treatment in children with CP has been shown in several studies [14, 15], and the results of the present study also showed that rehabilitation treatment is now being provided at a younger age in Korea.

In Korea, rehabilitation treatment of children with CP is performed on either an inpatient or outpatient basis. The results of this study show that the number of outpatients is higher than that of inpatients. The number of children receiving inpatient or outpatient rehabilitation therapy was the highest at the age of 4–5 years, while the cost of therapy increased until 6 years of age, after which it remained consistent. Additionally, the number of treatments per person increased gradually with age. This suggests that children who continue to receive rehabilitation therapy after the age of 4–5 years may experience CP at a moderate to severe degree of disability.

In the case of BTx., it can be administered to children with CP from the age of two years and higher under the current health insurance benefit standard. When we evaluated the changes in the time of onset of treatment, it was revealed that BTx. begins at a younger age than before. Recently, BTx. started being performed immediately after a child with CP reaches the age of two years, which is the minimum age for which the insurance benefit is given. Compared to children born in 2004, the average number of cases for BTx. per year was higher in children born in 2007. BTx. has been shown to be effective in the treatment of spasticity in children with CP [3], and it is evident that BTx. is increasingly being used as one of the primary treatment methods for pediatric CP in Korea. It is known that neutralizing antibodies are one of the causes of the secondary nonresponder phenomenon, i.e., the therapy was effective in the first treatment of BTx. but not effective for subsequent treatment. As such, it is recommended that the injection interval be at least three months to reduce the risk of antibody formation [16]. In the present study, even in the year when the number of BTx. treatments per person per year was the greatest, the average number of treatments was 2.4 with the interval being at least five months. Therefore, the treatment is being carried out within the provided guidelines.

Lastly, there was no difference in the cost of rehabilitation therapy based on the economic level of the family. This is consistent with the findings of a Canadian study [17] conducted in a setting in which a public health care system operated. Under Korea National Health Insurance, there is an expenditure portion the individual has to pay as copayments; however, it seems that there was no difference according to the income level because the portion covered by the NHIS was large. Only the therapies covered by health insurance were included in this study. However, because the costs of private centers, vouchers, and noninsurance-covered treatments such as speech and language therapy, cognitive therapy, intrathecal baclofen pump, and robot-assisted gait therapy were not included, it was difficult to interpret the differences in actual treatment cost according to income level.

### Study limitations

The limitations of this study were that first, only rehabilitation treatments under the benefit of the NHIS were analyzed. Second, alcohol or phenol nerve block, orthopedic surgery and selective dorsal rhizotomy cases were not included in the analysis because the number of cases was too small. Finally, we could not make a detailed comparison according to the severity of disease.

## Conclusions

As a result of using the NHID, it was recently found that rehabilitation therapy and spasticity treatment of children with CP are being performed increasingly earlier in Korea. In addition, it was confirmed that the nature of rehabilitation treatment for children with CP differed according to age. For the numbers of children receiving rehabilitation therapy and BTx. were highest at 1–5 years of age and 6–7 years of age, respectively. The expenditure for rehabilitation therapy gradually increased until six years of age, after which a similar level was maintained. Furthermore, the cost of rehabilitation therapy was not affected by the economic level of the family. Based on these results, more sophisticated health policies that consider age will be needed to provide rehabilitation for children with CP.

## Abbreviations

BTx: Botulinum toxin injection
CP: Cerebral palsy
